# Structural and Vascular Features of Macula Related to the Recurrence of Macular Edema in Central Retinal Vein Occlusion After Anti-VEGF Therapy

**DOI:** 10.1155/joph/8824342

**Published:** 2025-02-28

**Authors:** Dazhuang Ren, Cece Zhao, Gaoxu Wei, Xiaoyun Hou, Chang Li, Zhiqing Li

**Affiliations:** Tianjin Key Laboratory of Retinal Functions and Diseases, Tianjin Branch of National Clinical Research Center for Ocular Disease, Eye Institute and School of Optometry, Tianjin Medical University Eye Hospital, Tianjin, China

## Abstract

**Purpose:** To identify the structural and vascular features of the macula related to the recurrence of macular edema (ME) in central retinal vein occlusion (CRVO) after intravitreal anti-VEGF injections.

**Methods:** This was a cross-sectional study including CRVO patients without ME and age-matched individuals. CRVO patients were divided into the ME-resolved group and the ME-recurrence group on the basis of whether ME recurred within 3 months. All subjects provided a detailed history and underwent a comprehensive ophthalmological examination. Measurements of the macula by swept-source optical coherence tomography angiography (SS-OCTA) were recorded. We also created the Δparameter, which represents the difference in OCTA parameters between CRVO-affected eyes and their fellow eyes.

**Results:** The study included 13 ME-resolved CRVO patients, 20 ME-recurrence CRVO patients, and 24 age-matched controls. Compared with the ME-recurrence group, the ME-resolved group had a longer CRVO duration, more previous intravitreal anti-VEGF injections, and a higher proportion of previous retinal photocoagulation (all *p* < 0.05). Additionally, retinal thickness (RT) and choroidal thickness (CT) were thinner in the ME-resolved group than in the ME-recurrence and control groups (all *p* < 0.01). The ME-resolved group also had significantly lower vessel density (VD) in both superficial and deep vascular complexes (SVC/DVC) and larger foveal avascular zone area (FAZa) in SVC and DVC than the ME-recurrence group and the control group (all *p* < 0.01). The results were the same with the Δparameters. Multivariate logistic regression revealed that ΔVD and ΔFAZa in SVC and DVC were independently associated with ME recurrence after adjusting for the effects of CRVO duration, previous anti-VEGF injections, and retinal photocoagulation (all *p* < 0.05).

**Conclusion:** With prolonged CRVO duration, more anti-VEGF injections, and more retinal photocoagulation procedures, retinal, choroidal, and vascular atrophy in the macula occurs in CRVO eyes, making ME less likely to recur. Macular vascular atrophy is vital for the resolution of ME and might be a manifestation of capillary remodeling.

## 1. Introduction

Central retinal vein occlusion (CRVO) is a prevalent retinal vascular disease, with a prevalence ranging from 0.1% to 0.4% [1, 2]. CRVO often leads to severe visual impairment. Macular edema (ME) is the most common complication and the primary cause of visual impairment in patients with CRVO [3]. The clinical application of anti-VEGF therapy has improved the prognosis of CRVO, effectively reducing ME and improving visual function [4]. However, the recurrence of ME is a common complication following anti-VEGF therapy [5]. While some patients achieve sustained resolution of ME after several anti-VEGF injections, others experience recurrences shortly after the injections. This discrepancy suggests potential underlying structural or vascular differences in the fundus between these two groups of CRVO patients.

Previous studies have indicated that ME in retinal vascular diseases is associated with macular ischemia and disruption of the blood–retinal barrier [6]. Optical coherence tomography angiography (OCTA) studies on CRVO have revealed a reduction in the vessel density (VD) of the macula, suggesting compromised retinal perfusion [7]. Therefore, we hypothesized that the recurrence of ME in CRVO might be influenced by several factors, such as retinal metabolic oxygen demand, the degree of retinal ischemia, and the compensatory oxygen supply capacity of the choroid.

In this study, we used swept-source optical coherence tomography angiography (SS-OCTA) to compare the macular structure and vascular network among ME-resolved CRVO patients, ME-recurrence CRVO patients, and age-matched individuals. In this way, we would be able to identify potential biomarkers associated with the stability of CRVO and gain further insight into the mechanism underlying the recurrence of ME in CRVO.

## 2. Materials and Methods

This cross-sectional study adhered to the tenets of the Declaration of Helsinki and was approved by the Ethics Committee of Tianjin Medical University Eye Hospital (2024KY(L)-09).

### 2.1. Subjects and Inclusion/Exclusion Criteria

We included patients who were diagnosed with CRVO and age-matched individuals who visited the Medical Retina and Neuro-Ophthalmology Department of Tianjin Medical University Eye Hospital from December 2022 to May 2024.

The study inclusion criteria for CRVO patients were as follows: (1) unilateral CRVO confirmed by a senior retinal specialist (Z.L.) by multimodal imaging; (2) the patient had completed a course of 3 monthly loading anti-VEGF injections with ranibizumab (Lucentis) followed by a pro re nata (PRN) regimen using either ranibizumab (Lucentis) or conbercept (Lumitin); (3) this visit was conducted 1 month after the most recent injection; and (4) OCT B-scan showed no ME at this visit. CRVO patients were assigned to the ME-resolved group or the ME-recurrence group on the basis of whether OCT B-scan showed recurrent ME within 3 months after this visit. The control group included age-matched individuals who visited the Medical Retina and Neuro-Ophthalmology Department for routine examination. For each control, we randomly selected one of the two eyes.

Patients who were lost to follow-up within three months after this visit or who had previously received intravitreal steroid injections were excluded. Other exclusion criteria were as follows: (1) other fundus conditions except for mild diabetic retinopathy; (2) a signal strength index of OCTA image less than 7/10; (3) high myopia (spherical equivalent of -6D or more and myopic fundus changes); or (4) previous intraocular surgeries except for cataract surgery.

### 2.2. Ophthalmic Examination and Baseline Characteristics

All subjects underwent a comprehensive ophthalmic examination, including best-corrected visual acuity (BCVA), intraocular pressure (IOP) measurement, slit-lamp biomicroscopy (SL-D701, Topcon), dilated fundus examination, and SS-OCT/OCTA examination (VG200, Intalight). BCVA was evaluated with the Snellen visual chart and converted to the logarithm values of the minimum angle of resolution (logMAR). Other baseline characteristics such as age, sex, laterality, CRVO duration, number of previous intravitreal anti-VEGF injections, retinal photocoagulation history, and the presence of systemic disorders such as hypertension and diabetes were also recorded.

### 2.3. OCTA

The OCT/OCTA scan protocol involved a 6 × 6 mm^2^ scan area centered on the fovea. Vessel density in superficial vascular complex (VD-SVC) and vessel density in deep vascular complex (VD-DVC), retinal thickness (RT), choroidal thickness (CT), and ganglion cell–inner plexiform layer (GCIPL) thickness were automatically measured by the built-in algorithm. Additionally, we manually measured foveal avascular zone area (FAZa) with ImageJ software (https://imagej.net/, provided in the public domain by the National Institutes of Health, Bethesda, MD, USA).

**Figure 1:**
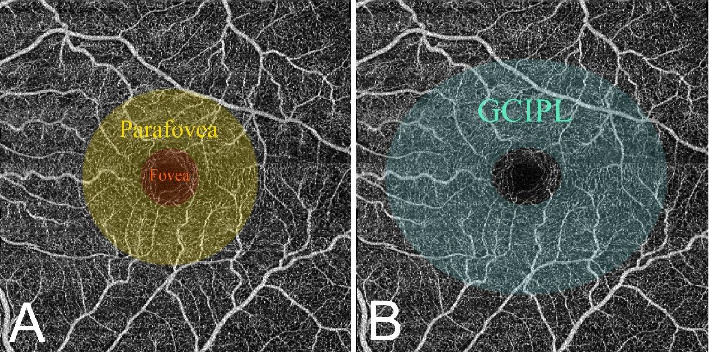
Segmentation of macula for measurements. (A) Segmentation of macula based on ETDRS grid. (B) Segmentation of macula for measuring GCIPL.

For measurement, the macular area was segmented into two concentric circles with diameters of 1 mm and 3 mm according to Early Treatment Diabetic Retinopathy Study (ETDRS) grid (Figure 1(A)). The inner circle and the outer annulus were designated as the rings of fovea and parafovea, respectively. VD-SVC, VD-DVC, RT, and CT were automatically measured based on this grid through the built-in algorithm. Superficial vascular complex (SVC) was defined as the vasculature extending from 5 μm above the inner limiting membrane to the interface between the inner two-thirds and outer one-third of the ganglion cell layer. Deep vascular complex (DVC) was defined as the vasculature extending from the interface to 25 μm below the lower border of the inner nuclear layer [8]. GCIPL was measured on the basis of an elliptical annulus. The vertical radii of the annulus were 0.5 and 2.0 mm, whereas the horizontal radii were 0.6 and 2.4 mm (Figure 1(B)) [9].

FAZa was manually measured in both SVC and DVC layers with ImageJ software. The observer manually delineated the boundary of the FAZ. The number of pixels within the FAZ was then converted to millimeters squared. Two experienced ophthalmologists who were blinded to the subjects' clinical information performed the measurements. For each image, the ophthalmologist measured the FAZa three times, and the mean of these measurements was used for analysis.

### 2.4. Delta OCTA Parameters

To further validate our findings, we introduced new parameters: ΔVD-SVC, ΔVD-DVC, ΔRT, ΔCT, ΔGCIPL, ΔFAZa-SVC, and ΔFAZa-DVC. These parameters represented the differences between the CRVO eye and its fellow eye and were calculated as the OCTA measurements of the CRVO eye minus those of the fellow eye.

### 2.5. Statistical Analysis

All the statistical analyses were conducted with SPSS 23.0. Quantitative data following a normal distribution are presented as mean ± standard deviation. Comparisons among three groups and between two groups used one-way analysis of variance (ANOVA) and Student's *t*-test, respectively. Quantitative data not following a normal distribution are presented as median (*p*25%-*p*75%), and the Kruskal–Wallis test was used for comparisons. Categorical data are presented as percentages, and the chi-square test was used for comparisons. Bonferroni correction or Tamhane's T2 test was used for all pairwise post hoc analysis. Intraclass correlation coefficient (ICC) was calculated to evaluate interobserver and intraobserver agreement for manually measured parameters. Multivariate logistic regression analyses between the ME-resolved group and the ME-recurrence group were conducted to reduce confounding bias caused by CRVO duration, previous intravitreal anti-VEGF injections, and retinal photocoagulation history. A *p* value of less than 0.05 was considered statistically significant.

## 3. Results

### 3.1. Demographic and Clinical Information

In total, 57 subjects, comprising 13 CRVO patients in the ME-resolved group, 20 CRVO patients in the ME-recurrence group, and 24 age-matched individuals in the control group, were included in this study.

The mean ages of the patients in the ME-resolved, ME-recurrence, and control groups were 63.9 ± 10.9 years, 58.8 ± 14.0 years, and 56.1 ± 12.2 years, respectively. BCVA in the control group was better than that in the ME-recurrence group, and BCVA in the ME-recurrence group was better than that in the ME-resolved group (ME-resolved: 0.52 ± 0.39 logMAR, ME-recurrence: 0.20 ± 0.21 logMAR, control: 0.01 ± 0.03 logMAR, *p* < 0.001). The ME-resolved group had a significantly longer CRVO duration than the ME-recurrence group (ME-resolved: 13.3 ± 5.9 months, ME-recurrence: 8.5 ± 2.9 months, *p*=0.003). Compared with the ME-recurrence group, the ME-resolved group received more previous intravitreal anti-VEGF injections (ME-resolved: 6.9 ± 2.7, ME-recurrence: 5.1 ± 1.4, *p*=0.015). Regarding retinal photocoagulation history, the proportion of patients with previous retinal photocoagulation in the ME-resolved group was significantly higher than that in the ME-recurrence group (ME-resolved: 46.2%, ME-recurrence: 10%, *p*=0.035). There were no significant differences in age, sex, laterality, IOP, or systemic disorders among the three groups. The details of demographic and clinical information are shown in Table 1.

### 3.2. RT, GCIPL Thickness, and CT

Regarding RT in all subregions, RT in the ME-resolved group was thinner than that in the control group, and RT in the control group was thinner than that in the ME-recurrence group (all *p* < 0.05). Compared with the ME-recurrence and control groups, the ME-resolved group had significantly thinner GCIPL (*p* < 0.05). There was no significant difference in GCIPL between the ME-recurrence group and the control group (*p* > 0.05). For CT in all subregions, the ME-resolved group was thinner than the ME-recurrence and control groups (all *p* < 0.05). No significant differences between the ME-recurrence group and the control group were found in any subregion of CT (all *p* > 0.05). The details of RT, GCIPL, and CT are shown in Table 2.

### 3.3. VDs in SVC and DVC

For VD-SVC in all subregions, ME-resolved group was lower than the ME-recurrence and control groups (all *p* < 0.001). There were no significant differences in VD-SVC between the ME-recurrence group and the control group (all *p* > 0.05). Regarding VD-DVC in fovea, the ME-resolved group was lower than the ME-recurrence and control groups (*p* < 0.001). There was no significant difference in VD-DVC in fovea between the ME-recurrence group and the control group (*p*=1.000). Regarding VD-DVC in parafovea and the whole region, the ME-resolved group was lower than the ME-recurrence group, and the ME-recurrence group was lower than the control group (all *p* < 0.01). The details of VD-SVC and VD-DVC among the three groups are shown in Table 3.

### 3.4. FAZa in SVC and DVC

The measurements of FAZa-SVC taken by the two observers showed excellent intraobserver (observer1: ICC = 0.987, 95% CI = 0.982–0.991, *p* < 0.001; observer2: ICC = 0.980, 95% CI = 0.971–0.986, *p* < 0.001) and interobserver (ICC = 0.960, 95% CI = 0.940–0.974, *p* < 0.001) agreement. FAZa-DVC also showed excellent intraobserver (observer1: ICC = 0.987, 95% CI = 0.981–0.991, *p* < 0.001; observer2: ICC = 0.984, 95% CI = 0.977–0.989, *p* < 0.001) and interobserver (ICC = 0.967, 95% CI = 0.950–0.978, *p* < 0.001) agreement.

**Figure 2:**
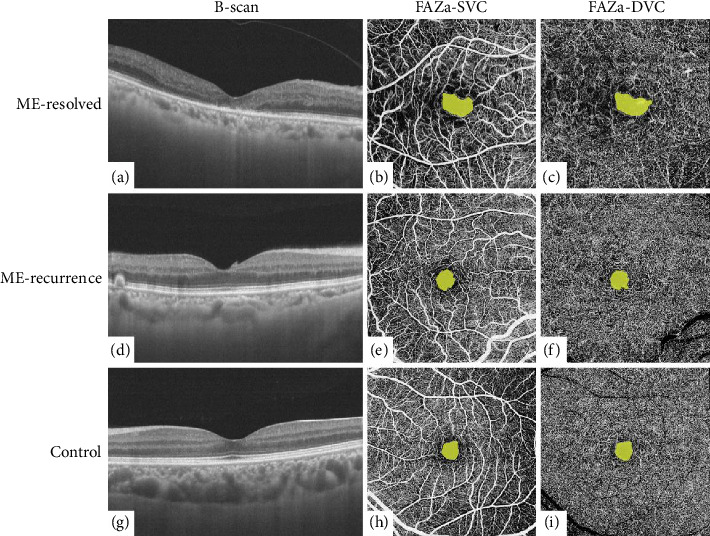
Representative B-scan and FAZa images of the ME-resolved, ME-recurrence, and control groups. (a, d, g) B-scans of the three groups. (b, e, h) FAZa-SVC images of the three groups. (c, f, i) FAZa-DVC images of the three groups. FAZa-DVC, foveal avascular zone area in deep vascular complex; FAZa-SVC, foveal avascular zone area in superficial vascular complex.

Compared with the ME-recurrence and control groups, the ME-resolved group had significantly larger FAZa-SVC and FAZa-DVC (all *p* < 0.01). There were no significant differences in FAZa-SVC or FAZa-DVC between the ME-recurrence group and the control group (all *p* > 0.05). Representative B-scan and FAZa images of the three groups are shown in Figure 2. The details of FAZa-SVC and FAZa-DVC are shown in Table 4.

### 3.5. Delta Parameters Between ME-Resolved Group and ME-Recurrence Group

**Figure 3:**
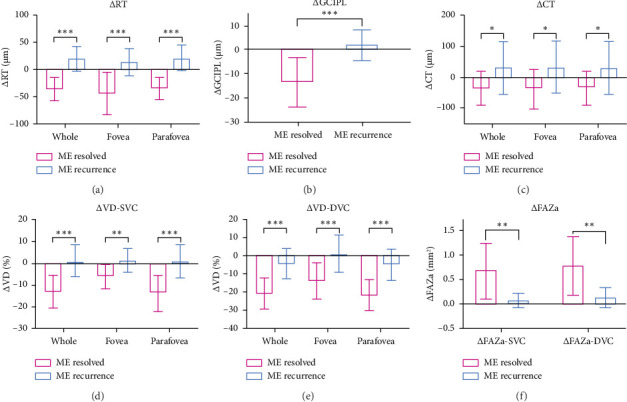
Comparisons of ΔRT, ΔGCIPL, ΔCT, ΔVD-SVC, ΔVD-DVC, and ΔFAZa between the ME-resolved group and the ME-recurrence group. (a) ΔRT, Δretinal thickness. (b) ΔGCIPL, Δganglion cell–inner plexiform layer thickness. (c) ΔCT, Δchoroidal thickness. (d) ΔVD-SVC, Δvessel density in superficial vascular complex. (e) ΔVD-DVC, Δvessel density in deep vascular complex. (f) ΔFAZa, Δfoveal avascular zone area. ^∗^*p* < 0.05; ⁣^∗∗^*p* < 0.01; ⁣^∗∗∗^*p* < 0.001.

For all subregions of ΔRT, ΔGCIPL, and ΔCT, the ME-resolved group was significantly lower than the ME-recurrence group (all *p* < 0.05, Figures 3(a), 3(b), and 3(c)). Differences in ΔVD-SVC and ΔVD-DVC were also significant, with ΔVD-SVC and ΔVD-DVC in all subregions being lower in the ME-resolved group than in the ME-recurrence group (all *p* < 0.01, Figures 3(d) and 3(e)). Regarding ΔFAZa-SVC and ΔFAZa-DVC, the ME-resolved group was higher than the ME-recurrence group (all *p* < 0.01, Figure 3(f)).

### 3.6. Multivariate Logistic Regression Analysis

We conducted multivariate logistic regression analyses to exclude the influences of CRVO duration, previous intravitreal anti-VEGF injections, and previous retinal photocoagulation on OCTA parameters. We included ΔVD-SVC, ΔVD-DVC, ΔRT, ΔGCIPL, ΔCT, ΔFAZa-SVC, and ΔFAZa-DVC separately with the number of previous intravitreal anti-VEGF injections, previous retinal photocoagulation, and CRVO duration in multivariate logistic regression analyses. The results showed that ΔVD-SVC, ΔVD-DVC, ΔFAZa-SVC, ΔFAZa-DVC, and ΔGCIPL were significant (all *p* < 0.05). ΔRT and ΔCT were more strongly associated with CRVO duration, number of previous intravitreal anti-VEGF injections, and previous retinal photocoagulation (all *p* > 0.05). The details of multivariate logistic regression analyses are shown in Table 5.

## 4. Discussion

With the clinical application of intravitreal anti-VEGF therapy, there has been revolutionary progress in the treatment of ME caused by CRVO. Large-scale clinical trials have demonstrated that intravitreal anti-VEGF injections can effectively reduce ME and improve visual acuity [10–13]. However, many patients experienced frequent ME recurrence within a few months after intravitreal anti-VEGF injections and required more injections [14–16]. Therefore, identifying the factors related to ME recurrence is important, as it can help clinicians predict disease progression and further understand the underlying mechanism.

In the pathogenesis of ME in retinal vein occlusion (RVO), after retinal ischemia and hypoxia caused by vein occlusion, pericyte loss develops, and the levels of cytokines such as VEGF increase [17, 18]. This process disrupts inner blood–retinal barrier (iBRB), resulting in vascular dilation and leakage, which ultimately triggers ME [6]. Our study found that RT in the ME-recurrence group was thicker than that in the control group, whereas RT in the ME-resolved group was thinner than that in the control group. Our results for ΔRT also supported this finding. The results for ΔRT showed that ME-resolved eyes had thinner RT compared to their fellow eyes, whereas ME-recurrence eyes had thicker RT. Our findings indicated that ME-recurrence eyes exhibited vascular leakage and had already developed subclinical ME, which could not be identified through morphology. ME-recurrence eyes might have high VEGF levels. Previous studies on branch retinal vein occlusion (BRVO) also showed that retinal thickening was associated with ME recurrence [19–21]. Our results are consistent with these studies. ME-resolved CRVO eyes exhibited retinal atrophy with minimal vascular leakage. These findings suggest that ME-resolved eyes are relatively stable and might have low VEGF levels. In addition, we found that GCIPL was thinner in the ME-resolved group than in the ME-recurrence and control groups. Our results for ΔGCIPL confirmed this trend. Similar results were reported in previous studies on BRVO. Zheng et al. reported GCIPL thinning in BRVO eyes with resolved ME [22]. These findings indicate that ME-resolved CRVO eyes indeed exhibited GCIPL atrophy and did not have edema.

Our study revealed that CT in the ME-resolved group was thinner than that in the ME-recurrence and control groups. Furthermore, our results for ΔCT showed that CT in ME-resolved eyes was thinner than that in fellow eyes, whereas CT in ME-recurrence eyes was thicker than that in fellow eyes. Our findings indicate that ME-recurrence eyes had relative choroidal edema, whereas ME-resolved eyes had choroidal atrophy. Similar results were reported in studies on BRVO and diabetic macular edema (DME). Okamoto et al. reported that BRVO eyes with resolved ME had thinner CT than BRVO eyes with recurrent ME [23]. Mathis et al. reported that DME eyes with a relatively thick choroid tended to develop recurrent DME [24]. Additionally, previous studies showed that CT decreased after intravitreal anti-VEGF treatment but increased before the recurrence of ME [25–27]. These findings suggest that VEGF levels might influence CT, with high VEGF levels potentially causing choroidal thickening. In our study, thicker CT in ME-recurrence eyes might indicate higher VEGF levels, whereas thinner CT in ME-resolved eyes might indicate lower VEGF levels.

For VD-SVC and VD-DVC, our study showed that both VD-SVC and VD-DVC were lower in the ME-resolved group than in the ME-recurrence and control groups. Our findings suggest that ME-resolved eyes had macular vascular atrophy. Similar results were reported in previous BRVO studies, where BRVO eyes with lower macular VD were less likely to experience ME recurrence [28, 29]. These results indicate that macular vascular atrophy might reduce the likelihood of ME recurrence. Schmid et al. reported that retinal vascular loss could lead to a reduction in retinal oxygen consumption [30]. A previous study on BRVO rat models also revealed that capillary dropout developed in the late stage [31]. These findings suggest that macular vascular atrophy might be a manifestation of capillary remodeling and cause a reduction in oxygen demand, less VEGF production, and thus less vascular leakage. Our results for FAZa also supported this hypothesis. ME-resolved eyes showed smaller FAZa in both SVC and DVC compared to ME-recurrence eyes and normal eyes. Similar findings were also reported in previous BRVO studies. Tomita et al. found that FAZa in ME-resolved eyes was larger than that in ME-recurrence eyes [32]. Tripathy et al. reported that FAZa enlargement was associated with capillary remodeling [33]. Thus, macular vascular atrophy might be a manifestation of capillary remodeling. ME-resolved eyes might have completed capillary remodeling, whereas ME-recurrence eyes might not have completed capillary remodeling.

The results for ΔVD and ΔFAZa in the ME-recurrence group revealed more details. In the ME-recurrence group, ΔVD and ΔFAZa in SVC were close to zero, whereas ΔVD and ΔFAZa in DVC were greater than zero. This finding is also consistent with our VD-SVC and VD-DVC findings, where VD-SVC in the ME-recurrence group was similar to that in the control group, and VD-DVC was lower in the ME-recurrence group than in the control group. These findings indicate that macular vascular atrophy was more pronounced in the deep layer than in the superficial layer. Previous studies on BRVO also revealed that the reduction rate of VD in the deep layer was higher than in the superficial layer [34, 35]. These results suggest that capillary remodeling might initiate in the deep layer and progress until completed in the superficial layer, at which point CRVO tends to stabilize.

Our study found that BCVA was better in the ME-recurrence group than in the ME-resolved group. This could be related to the severe macular atrophy observed in ME-resolved eyes. In contrast, ME-recurrence eyes had only mild subclinical ME and the layers of the macula remained relatively intact. In our study, RT in the ME-resolved group was quite a bit thinner than that in the control group, and FAZa in the ME-resolved group was notably larger than that in the control group. In contrast, RT in the ME-recurrence group was slightly thicker than that in the control group, and FAZa was not significantly larger. These findings indicated severe macular atrophy in ME-resolved eyes, whereas macular damage in ME-recurrence eyes was relatively mild. These differences might explain why the ME-recurrence group exhibited better BCVA compared to the ME-resolved group. Similar results were reported in other studies on ME. Marolo et al. reported that in DME patients, greater deviation from normal RT, whether thicker or thinner, was associated with worse BCVA [36]. Additionally, previous studies on DME and CRVO also showed that larger FAZa was associated with worse BCVA [37, 38]. Therefore, structural and vascular atrophy might contribute to worse BCVA in ME-resolved eyes.

Compared with those in the ME-recurrence group, CRVO duration was longer, the number of intravitreal anti-VEGF injections was higher, and the proportion of eyes with previous retinal photocoagulation was higher in the ME-resolved group. To exclude the potential influence of these factors on OCTA parameters, we included these factors and each Δparameter separately in multivariate logistic regression analyses. Our results indicated that RT and CT were more related to CRVO duration, previous intravitreal anti-VEGF injections, and previous retinal photocoagulation, whereas FAZa, VD, and GCIPL were more related to ME recurrence. These indicate that the recurrence of ME is related to the macular vasculature. Studies on RVO reported that CT and RT were related to disease duration and previous intravitreal anti-VEGF treatments [25, 39]. A study on BRVO also revealed that macular VD was not associated with disease duration [33]. Our findings are consistent with those of these studies and further support that macular vascular atrophy indeed influences ME recurrence in CRVO.

The strength of our study was that we assessed the differences between ME-resolved eyes and ME-recurrence eyes at multiple levels. We not only compared CRVO eyes with normal eyes but also confirmed our findings through Δparameter. However, there are also several limitations in our study. First, CRVO patients without ME had a lower frequency of follow-up visits. This could cause selection bias. Second, we did not record types of anti-VEGF drugs in PRN regimen, whether ranibizumab or conbercept. Third, FAZa in SVC and DVC was manually measured and this might cause measurement error.

In conclusion, we found that with prolonged CRVO duration, more anti-VEGF injections, and more retinal photocoagulation procedures, CRVO eyes exhibited retinal, choroidal, and macular vascular atrophy, making ME less likely to recur. Macular vascular atrophy is vital for the resolution of ME and might be a manifestation of capillary remodeling.

## Figures and Tables

**Table 1:** Demographic and clinical information.

	A. ME-resolved	B. ME-recurrence	C. Control	*p* value
Age (years)	63.9 ± 10.9	58.8 ± 14.0	56.1 ± 12.2	0.204
Sex (M, %)	6 (46.2%)	11 (55.0%)	8 (33.3%)	0.347
Laterality (OD, %)	9 (69.2%)	8 (40.0%)	13 (54.2%)	0.254
BCVA (logMAR)	0.52 ± 0.39	0.20 ± 0.21	0.01 ± 0.03	< 0.001⁣^∗^
IOP (mmHg)	15.7 ± 2.3	16.1 ± 3.7	14.6 ± 3.5	0.355
Diabetes (+, %)	6 (46.2%)	2 (10.0%)	6 (25.0%)	0.062
HBP (+, %)	8 (61.5%)	11 (55.0%)	8 (33.3%)	0.182
CRVO duration (months)	13.3 ± 5.9	8.5 ± 2.9	/	0.016⁣^∗^
Number of previous IVIs	6.9 ± 2.7	5.1 ± 1.5	/	0.035⁣^∗^
Retinal photocoagulation (+, %)	6 (46.2%)	2 (10.0%)	/	0.035⁣^∗^

Abbreviations: BCVA, best-corrected visual acuity; CRVO, central retinal vein occlusion; HBP, high blood pressure; IOP, intraocular pressure; IVI, intravitreal injection.

^∗^ *p* < 0.05.

**Table 2:** Comparisons of RT, GCIPL, and CT among the ME-resolved, ME-recurrence, and control groups.

	A. ME-resolved	B. ME-recurrence	C. Control	*p* value	*p* value of post hoc comparisons		
					A versus B	A versus C	B versus C
RT (μm)							
Whole	285.3 ± 25.2	336.8 ± 25.0	315.3 ± 15.3	< 0.001⁣^∗^	< 0.001⁣^∗^	< 0.001⁣^∗^	0.005⁣^∗^
Fovea	216.1 ± 33.0	268.6 ± 32.9	247.4 ± 20.2	< 0.001⁣^∗^	< 0.001⁣^∗^	0.007⁣^∗^	0.049⁣^∗^
Parafovea	293.9 ± 25.7	345.3 ± 25.2	323.8 ± 15.5	< 0.001⁣^∗^	< 0.001⁣^∗^	0.001⁣^∗^	0.006⁣^∗^
GCIPL (μm)	63.2 ± 11.0	79.8 ± 7.9	78.9 ± 7.1	< 0.001⁣^∗^	< 0.001⁣^∗^	< 0.001⁣^∗^	1.000
CT (μm)							
Whole	261.1 ± 43.7	330.1 ± 102.2	341.5 ± 118.9	0.004⁣^∗^	0.037⁣^∗^	0.017⁣^∗^	0.981
Fovea	266.5 ± 44.5	338.6 ± 104.2	349.7 ± 118.9	0.003⁣^∗^	0.032⁣^∗^	0.014⁣^∗^	0.983
Parafovea	260.5 ± 43.8	329.1 ± 102.0	340.5 ± 118.9	0.004⁣^∗^	0.039⁣^∗^	0.018⁣^∗^	0.981

*Note:* CT, macular choroidal thickness.

Abbreviations: GCIPL, ganglion cell–inner plexiform layer; RT, retinal thickness.

^∗^ *p* < 0.05.

**Table 3:** Comparisons of VD-SVC and VD-DVC among the ME-resolved, ME-recurrence, and control groups.

	A. ME-resolved	B. ME-recurrence	C. Control	*p* value	*p* value of all pairwise post hoc comparisons		
					A versus B	A versus C	B versus C
VD-SVC (%)							
Whole	32.30 ± 9.52	48.96 ± 6.08	48.78 ± 4.82	< 0.001⁣^∗^	< 0.001⁣^∗^	< 0.001⁣^∗^	0.999
Fovea	3.59 ± 3.54	12.78 ± 5.69	11.15 ± 4.78	< 0.001⁣^∗^	< 0.001⁣^∗^	< 0.001⁣^∗^	0.826
Parafovea	35.89 ± 10.39	53.49 ± 6.37	53.48 ± 5.06	< 0.001⁣^∗^	< 0.001⁣^∗^	< 0.001⁣^∗^	1.000
VD-DVC (%)							
Whole	30.29 ± 9.90	46.60 ± 5.67	52.86 ± 3.08	< 0.001⁣^∗^	< 0.001⁣^∗^	< 0.001⁣^∗^	< 0.001⁣^∗^
Fovea	7.91 ± 9.16	23.49 ± 10.70	23.13 ± 7.67	< 0.001⁣^∗^	< 0.001⁣^∗^	< 0.001⁣^∗^	1.000
Parafovea	33.09 ± 10.20	49.49 ± 5.43	56.58 ± 2.78	< 0.001⁣^∗^	0.004⁣^∗^	< 0.001⁣^∗^	< 0.001⁣^∗^

Abbreviations: VD-DVC, vessel density in deep vascular complex; VD-SVC, vessel density in superficial vascular complex.

^∗^ *p* < 0.05.

**Table 4:** Comparisons of FAZa-SVC and FAZa-DVC among the ME-resolved, ME-recurrence, and control groups.

	A. ME-resolved	B. ME-recurrence	C. Control	*p* value	*p* value of all pairwise post hoc comparisons		
					A versus B	A versus C	B versus C
FAZa-SVC (mm^2^)	1.01 ± 0.60	0.40 ± 0.18	0.36 ± 0.12	0.003⁣^∗^	0.010⁣^∗^	0.006⁣^∗^	0.728
FAZa-DVC (mm^2^)	1.07 ± 0.62	0.43 ± 0.21	0.34 ± 0.10	0.001⁣^∗^	0.009⁣^∗^	0.003⁣^∗^	0.227

Abbreviations: FAZa-DVC, foveal avascular zone area in deep vascular complex; FAZa-SVC, foveal avascular zone area in superficial vascular complex.

^∗^ *p* < 0.05.

**Table 5:** Multivariate logistic regression analysis of Δparameters separately with number of previous intravitreal anti-VEGF injections, previous retinal photocoagulation, and CRVO duration.

	OR	95% CI	*p* value
ΔRT	39.604	0.000–1.576 × 10^304^	0.992
ΔGCIPL	1.261	1.042–1.526	0.017⁣^∗^
ΔCT	1.011	0.999–1.028	0.069
ΔVD-SVC	1.512	1.088–2.101	0.014⁣^∗^
ΔVD-DVC	1.481	1.043–2.101	0.028⁣^∗^
ΔFAZa-SVC	0.007	0.000–0.770	0.007⁣^∗^
ΔFAZa-DVC	0.023	0.001–0.920	0.045⁣^∗^

*Note:* CT, macular choroidal thickness.

Abbreviations: FAZa-DVC, foveal avascular zone area in deep vascular complex; FAZa-SVC, foveal avascular zone area in superficial vascular complex; GCIPL, ganglion cell–inner plexiform layer; RT, retinal thickness; VD-DVC, vessel density of deep vascular complex; VD-SVC, vessel density of superficial vascular complex.

^∗^ *p* < 0.05.

## Data Availability

All data generated or analyzed during this study are included in this article. Further inquiries can be directed to the corresponding author.
